# Comprehensive characterization of the competitive endogenous RNA network revealing its immune-related functions in hepatic ischemia-reperfusion injury

**DOI:** 10.1371/journal.pone.0327101

**Published:** 2025-07-24

**Authors:** Lirong Liu, Qi Wang, Shuang Qin, Chang Su, Xin Hai, Zhenkuan Yuan

**Affiliations:** Department of Pharmacy, The First Affliated Hospital of Harbin Medical University, Harbin, China; Cedars-Sinai Medical Center, UNITED STATES OF AMERICA

## Abstract

Hepatic ischemia-reperfusion injury (HIRI) is a common complication in liver surgery and transplantation. Recent studies have revealed the significant role of the competing endogenous RNA (ceRNA) network in HIRI. Herein, we comprehensively analyzed the HIRI-related ceRNA network and its correlation with immune-related pathways and immune cells in HIRI patients. We identified 449 lncRNAs, 26 miRNAs, and 548 mRNAs differentially expressed in HIRI patients. We constructed a HIRI-related ceRNA network in liver transplant patients consisting of 3 lncRNAs, 3 miRNAs, and 29 mRNAs. Biological function analysis showed that the HIRI-related ceRNA network contributes to HIRI progression by regulating calcium ion-related regulatory pathways and processes. An immune-related ceRNA subnetwork, which consists of 1 lncRNA (PARD6G-AS1), 1 miRNA (hsa-miRNA-125b-5p), and 4 mRNAs (PLAU, CCR5, FGF5 and IL24) was obtained. The immune-related ceRNA subnetwork was significantly related to the immune-related pathways and immune cell infiltration. The *PARD6G-AS1*/*miR-125b-5p*/*IL24* axis was identified as a potential ceRNA sponge that may influence NK cell activity in HIRI. Our results underlined that the lncRNA-miRNA-mRNA ceRNA network can positively or negatively regulate immune-related functions and infiltrating immune cells mediated HIRI, which could provide further insight into novel molecular therapeutic targets.

## Introduction

Ischemia-reperfusion injury (IRI) is defined as increased cellular damage and death after the restoration of blood flow to previously ischemic tissues, resulting in metabolic dysfunction and structural damage [1]. Hepatic ischemia-reperfusion injury (HIRI) is an inevitable complication of hepatic surgery or transplantation, and drives graft rejection after liver transplantation, causing liver dysfunction or even failure [2]. However, since a systematic analysis of critical genes associated with the pathogenesis of HIRI is still lacking, it is not enough to elucidate the underlying pathological mechanism of HIRI.

More than 90% of the RNA transcripts in the human genome are non-coding RNAs (ncRNAs), including microRNAs and long noncoding RNAs (lncRNAs), which do not encode proteins but are critical for normal cell function and the development of many diseases [3]. MiRNAs are endogenous gene expression inhibitors that bind to the 3’UTR of target RNAs to inhibit translation of proteins or promote the degradation of mRNAs [4]. Numerous ncRNAs have been recently implicated as regulators in pathological states, such as HIRI. Moreover, increasing attention has also been paid to the regulatory networks of lncRNAs and miRNAs involved in HIRI, although the underlying molecular mechanisms remain incompletely understood. For example, Chen et al. found that during hepatectomy or transplantation, targeted blocking of *AK139328* might help alleviate HIRI [5]. According to Zhou et al., dexmedetomidine protected hepatocytes from reperfusion-induced damage and oxygen-glucose deprivation by enhancing *CCAT1* expression in vitro, and HIRI was mitigated by *CCAT1*, which increased cyclinD1 and reduced hepatocyte apoptosis in vitro [6]. In addition, several miRNAs could indicate the preoperative immune status of patients with liver transplant [7]. According to Farida et al, patients with acute renal failure after liver transplantation had significantly higher levels of *miR-122* and *miR-148a* [8]. MiRNA dysregulation was also related to cellular inflammation, oxidative stress, autophagy, and apoptosis of HIRI [9]. Moreover, Huang et al. demonstrated that lncRNA *MEG3* and *miR-34a* establish a mutual inhibitory feedback loop, proposing that the *MEG3*/*miR-34a*/*Nrf2* axis may serve as a potential therapeutic target for HIRI [10]. lncRNA *HOTAIR* might enhance autophagy of liver cells by the *miR-20b-5p*/*ATG7* axis and accelerate HIRI progression [11]. lncRNA *AK054386* has been indicated to function as a competitive endogenous RNA (ceRNA) for *miR-199*, thereby modulating endoplasmic reticulum stress (ERS)-related factors in HIRI [12]. The progression of HIRI is regulated by complex lncRNA-miRNA-mRNA networks involving their mutual activation and antagonism. A comprehensive understanding of lncRNA–miRNA interactions in HIRI is essential for the identification of novel therapeutic targets.

In addition, the immune system plays an important role in HIRI. The pathophysiological process of HIRI can be broadly distinguished into acute and chronic phases. Acute hepatocellular injury was accompanied by excessive production of free radicals, alongside macrophage and T-cell activation. Chronic hepatocellular injury was characterized by massive neutrophil infiltration, which contributed to the overproduction of cytokines and chemokines, as well as other leukocyte activation [13]. Although maintaining immune microenvironmental homeostasis is crucial, the comprehensive landscape of immune cell infiltration in HIRI remains incompletely elucidated.

**Fig 1:**
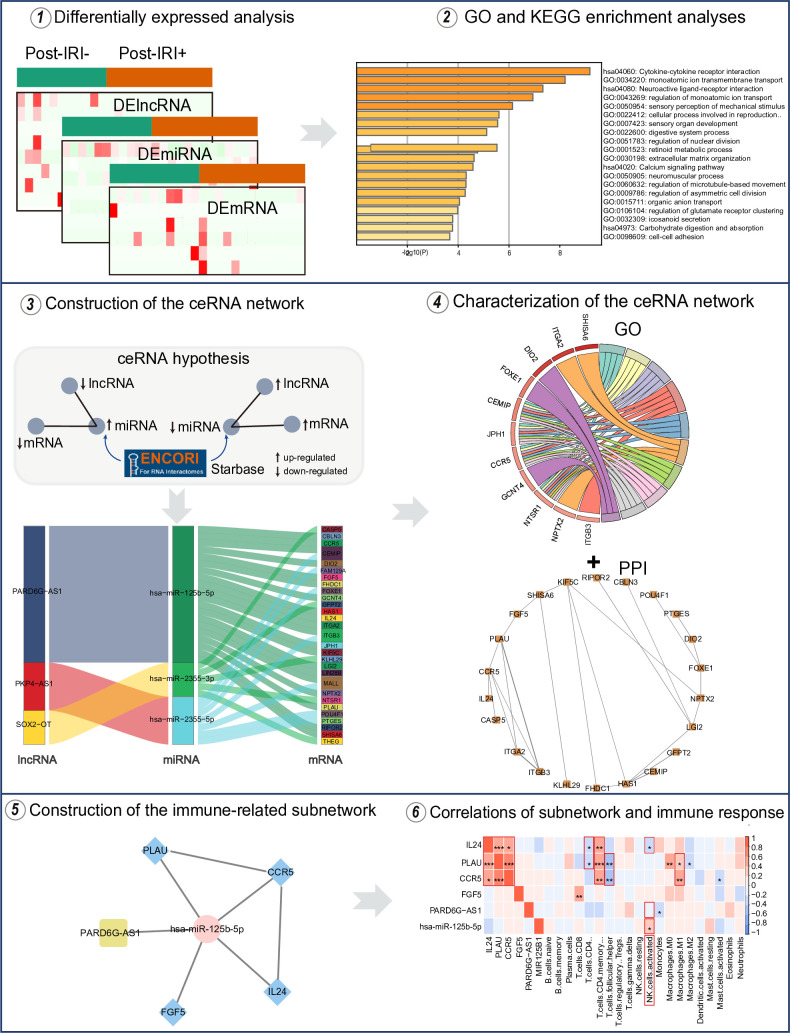
Flowchart of this study.

In this study, we identified differentially expressed (DE) lncRNAs, miRNAs and mRNAs between liver post-transplant patients with IRI+ and IRI- using data from the GEO database. Subsequently, we constructed a HIRI-related ceRNA network according to the ceRNA hypothesis. We comprehensively analyzed the ceRNA network, examined its correlation with immune-related pathways and immune cells in HIRI, and further identified an immune-related subnetwork. Our study aimed to understand the molecular regulatory network deeply underlying HIRI and to investigate the relationship between the ceRNA network and immune responses (Fig 1).

## Materials and methods

### Data preparation

In this study, GSE151648 datasets were downloaded from the Gene Expression Omnibus (GEO) database (https://www.ncbi.nlm.nih.gov/geo/). The GSE151648 dataset comprises RNA-seq gene expression profiles obtained from liver biopsy samples of 23 IRI+ and 17 IRI- patients after transplantation. The sequencing was performed using the Illumina HiSeq 3000 platform (GPL21290), aligned to the human reference genome hg38 [14].

### Identification of differentially expressed RNAs

To identify the differentially expressed genes (DEG) between IRI+ and IRI- after liver transplant, miRNAs, lncRNAs, and mRNAs were first annotated using the Gencode database [15]. HUGO Gene Nomenclature Committee (HGNC) was used to convert the miRNA symbols [16]. Differential expression analysis was subsequently conducted using the R package edgeR [17]. Differentially expressed lncRNA (DElncRNA), miRNA (DEmiRNA) and mRNA (DEmRNA) were identified using a threshold of absolute fold change (FC) > 2 and p-value < 0.05 [18].

### Functional annotation of DEGs

Metascape (http://metascape.org) is a web-based platform designed to provide a comprehensive, user-friendly suite of tools for the functional enrichment analysis of gene and protein lists, facilitating data-based decisions for the biomedical research community [19]. To investigate the biological functions of DEGs, we performed Gene Ontology (GO) and Kyoto Encyclopedia of Genes and Genomes (KEGG) pathway enrichment analyses using the Metascape platform.

### Construction of a ceRNA network

According to the competing endogenous RNA (ceRNA) hypothesis, lncRNAs can act as molecular sponges for miRNAs, thereby influencing post-transcriptional gene regulation. Two regulatory patterns were considered: the downregulated lncRNA–upregulated miRNA–downregulated mRNA axis and the upregulated lncRNA–downregulated miRNA–upregulated mRNA axis, reflecting potential ceRNA-mediated regulatory mechanisms. Accordingly, upregulated and downregulated miRNAs were mapped to the starBase database (http://starbase.sysu.edu.cn/) [20], whose corresponding interacting pairs of DElncRNA and DEmRNA were respectively identified based on the above regulatory paradigms to predict the ceRNA network [21]. To elucidate the regulatory functions of the ceRNA network, GO enrichment analysis was performed on the DEmRNAs involved in the network using the R package clusterProfiler [22].

### Integration of protein-protein interaction (PPI) network

Protein–protein interaction (PPI) analysis of the DEmRNAs within the ceRNA network was conducted using the STRING database (http://string-db.org/), applying a composite score threshold greater than 0.40 to identify significant interactions. Furthermore, the ceRNA network was visualized using Cytoscape software, and key hub genes were identified through module analysis using the Molecular Complex Detection (MCODE) plugin.

### Construction of immune-related ceRNA subnetwork

Firstly, miRNAs and lncRNAs related to 17 immune-related pathways were obtained, along with p-values of the correlation from the Regulon Atlas of Immune-related Pathways across Cancer Types (ImmReg) [23].

Subsequently, we acquired the immune-related pathways and immune cells from ImmLnc. ImmLnc is an integrative algorithm that comprehensively characterizes the lncRNA regulation landscape in the immunome [24]. ImmLnc also provides correlation coefficients and p-values quantifying the associations between lncRNAs and immune-related pathways or immune cells.

We then intersected the known immune-related genes from the ImmPort database with the mRNAs identified in the ceRNA network. ImmPort database (https://immport.niaid.nih.gov) is one of the largest open-access repositories of subject-level human immunology data, providing a comprehensive list of immunologically relevant genes curated with functions and Gene Ontology terms [25]. Finally, the immune-related ceRNA subnetwork was constructed according to the above immune-related RNA.

### Validation of the immunological relevance for the ceRNA subnetwork

CIBERSORT estimates the relative abundance and composition of immune cell types within a mixed cell population based on gene expression profiles [26]. Here, we evaluated the abundance of 22 immune cells in liver biopsy samples using CIBERSORT. Spearman’s correlation analysis was performed to investigate the relationships between members of the ceRNA network and immune cells.

## Result

### Differentially expressed RNAs between IRI+ and IRI- patients after liver transplant

**Fig 2:**
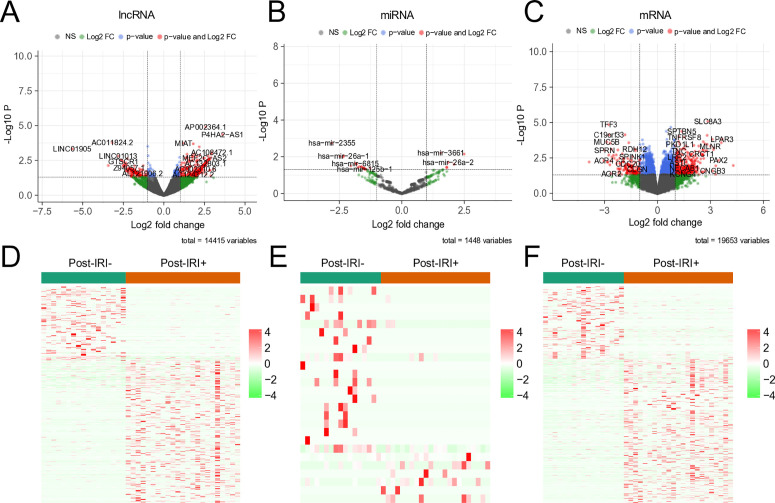
Identification of significant expression changes in RNAs after liver transplant. A-C. Volcano plot for the DElncRNAs, DEmiRNAs and DEmRNAs in GSE151648. The black dots and green represent genes that are not significantly differentially expressed between IRI+ samples and IRI- samples, and the red dots on the left represent the downregulated genes and on the right represent upregulated genes in IRI+ samples. D-F. Heatmap of the DElncRNAs, DEmiRNAs and DEmRNAs in GSE151648. Genes expressed at high levels are shown in red, and genes expressed at low levels are shown in green.

To determine the expression levels after liver transplant, we analyzed the expression of lncRNAs, miRNAs, and mRNAs in HIRI+ and HIRI- patients. Compared with the HIRI− group, the HIRI+ group exhibited significant differential expression, with 364 upregulated and 184 downregulated mRNAs, 294 upregulated and 155 downregulated lncRNAs, and 6 upregulated and 20 downregulated miRNAs (Fig 2).

### GO and KEGG pathway enrichment analyses of DEmRNAs

**Fig 3:**
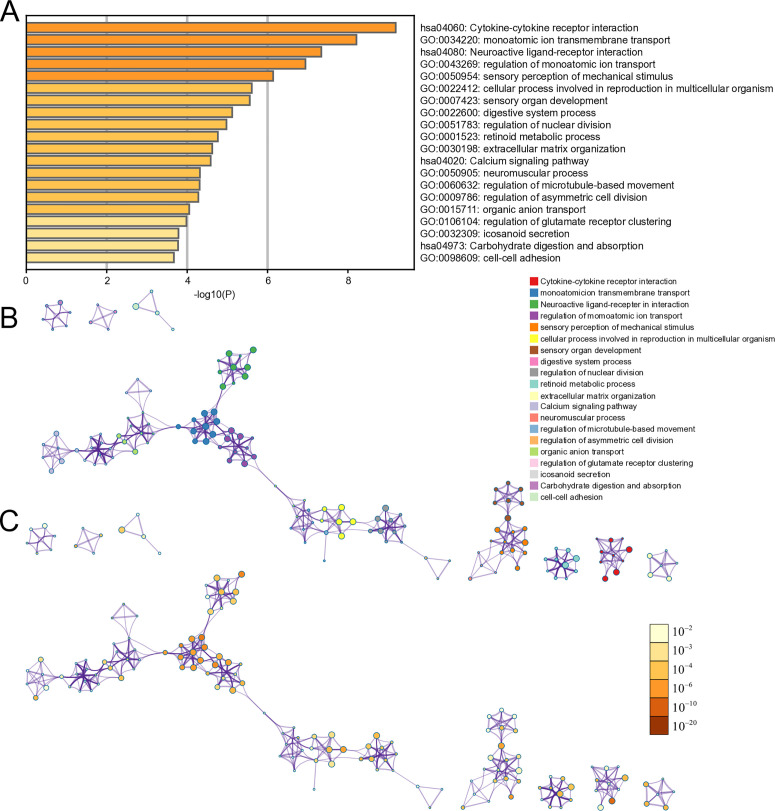
Functional enrichment analysis of 548 mRNAs. A. Bar graph of enriched terms across input gene lists colored by P values. B. Network of enriched terms colored by cluster ID, where nodes that share the same cluster ID are typically close to each other C. Network of enriched terms colored by P value, where terms containing more genes tend to have a more significant P value.

To explore the potential biological functions of the 548 DEmRNAs (p-values < 0.05), we conducted functional enrichment analysis and identified the top 20 GO biological processes and KEGG pathways representing the most statistically significant terms (Fig 3). The most significantly enriched GO term was monatomic ion transmembrane transport. Additionally, four key KEGG pathway clusters were identified: calcium signaling pathway, cytokine–cytokine receptor interaction, carbohydrate digestion and absorption, and neuroactive ligand–receptor interaction.

### Constructing the HIRI-related competitive endogenous RNA (ceRNA) network

**Fig 4:**
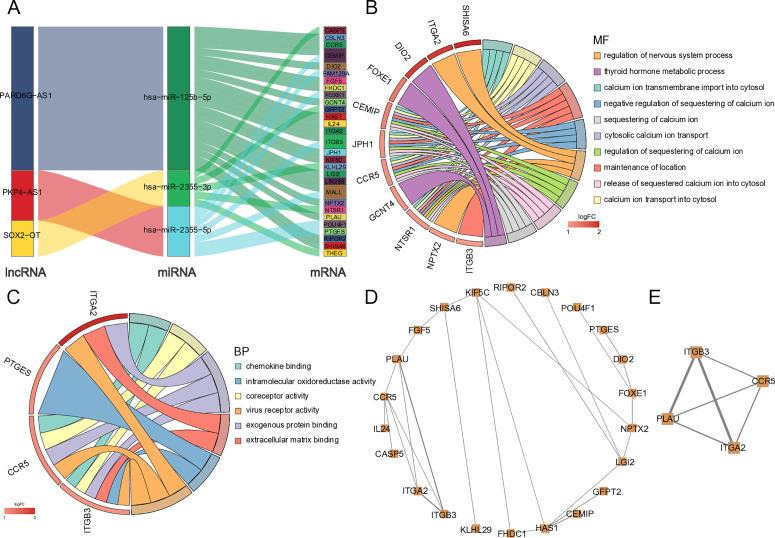
The functional annotation and protein-protein interaction (PPI) analysis of upmRNAs in the ceRNA network. A. ceRNA network constructed by upmRNAs, downmiRNAs, and uplncRNAs. B. The top 10 significantly enriched BP biological process and relevant genes. C. The significantly enriched MF biological process and relevant genes. D. PPI network of upmRNAs in the ceRNA network. E. Cluster analyzed by the MCODE in the whole PPI network.

We utilized the starBase database to predict the ceRNA network by mapping downregulated lncRNAs–upregulated miRNAs–downregulated mRNAs as well as upregulated lncRNAs–downregulated miRNAs–upregulated mRNAs, respectively. This analysis identified 32 upregulated lncRNA–downregulated miRNA–upregulated mRNA networks comprising three miRNAs (*hsa-miR-125b-5p*, *hsa-miR-2355-3p*, *hsa-miR-2355-5p*), three lncRNAs (*PARD6G-AS1*, *PKP4-AS1*, *SOX2-OT*), and 29 mRNAs (Fig 4A).

### Upregulated mRNAs from the ceRNA network participated in biological processes associated with IRI+ after liver transplantation

We subsequently retrieved the GO annotations of the upregulated mRNAs within the ceRNA network to assess their primary functional pathways. The analysis revealed 75 GO terms (adjusted P < 0.05), including 69 terms belonging to biological processes (BP) and 6 terms belonging to molecular functions (MF) (Fig 4B–C). Enrichment analysis of BP terms revealed a significant association with various calcium ion-related regulatory pathways and processes. For MF terms, chemokine binding and extracellular matrix binding were enriched. Previous studies have indicated that the regulation of ionized calcium homeostasis may become dysregulated during liver transplantation [27]. A study reported that the release of cytokines and chemokines could further aggravate the influence of IRI, in turn making the ischemia worse in the liver [28]. These results indicated that the ceRNA network could regulate IRI-related processes after the liver transplant.

### Protein interactions between upregulated mRNAs within the ceRNA network

The PPI network consisted of 22 nodes and 26 edges, suggesting direct or indirect protein regulatory relationships among the upregulated mRNAs within the ceRNA network (Fig 4D). Additionally, a module comprising CCR5, ITGB3, ITGA2, and PLAU was identified using MCODE (Fig 4E). Notably, the majority of these genes were involved in multiple IRI-related biological processes after the liver transplantation (Fig 4B-C).

### Immunological correlation analysis and construction of immune-related ceRNA subnetworks

Immune cells have been reported to play critical roles in exacerbating hepatic IRI-induced liver injuries, such as macrophages, Kupffer cells, lymphocytes, and neutrophils [29]. Immune cells, especially neutrophils, were activated and recruited into the liver after reperfusion, resulting in excessive neutrophil influx by positive feedback [30]. Neutrophil-formed extracellular traps (NETs) could exacerbate tissue inflammation and promote the development of HIRI [31].

**Fig 5:**
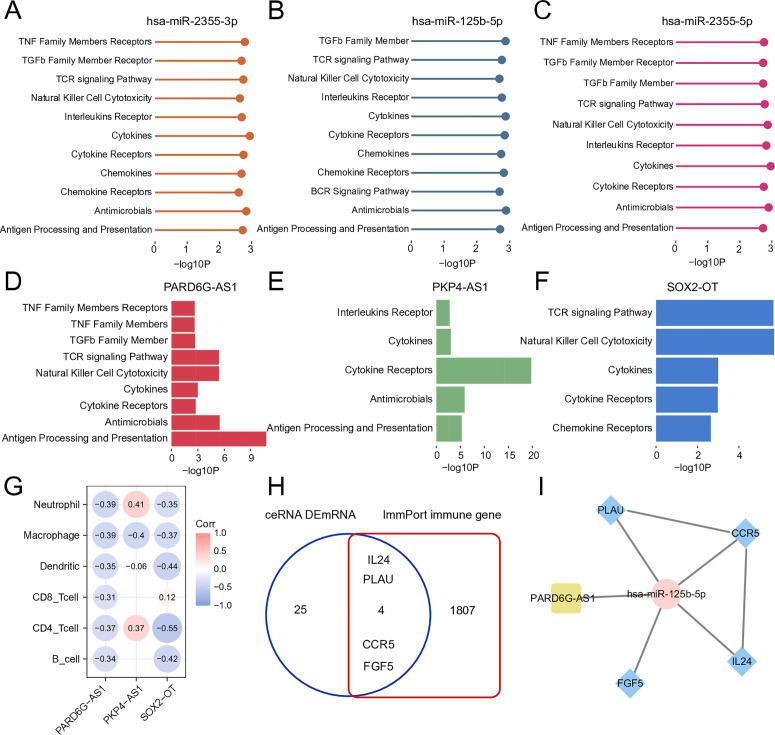
Correlation Analysis of RNA in ceRNA with immune pathways and immune cells, as well as the construction of ceRNA subnetwork. A-C. Lollipop chart showing the relationship between miRNAs in ceRNA and immune pathways. D-F. Bar Chart showing the relationship between lncRNAs in ceRNA and immune pathways. G. The relationship between lncRNAs in ceRNA and immune cells(P < 0.05). H. Venn diagram showing the Intersecting genes between mRNAs in ceRNA and known immune genes. I. ceRNA subnetwork constructed based on immune-related RNAs in ceRNA, the yellow node represents lncRNA, the pink node represents miRNA and the blue nodes represent mRNA, and the edges among mRNA are their PPI interactions.

Accordingly, we further screened for ceRNAs that may play regulatory roles in immune function associated with HIRI. We conducted a correlation analysis between the IRI-related ceRNA network and immune-related pathways, as well as immune cells. Our analysis revealed that lncRNAs and miRNAs within the IRI-associated ceRNA network were significantly linked to a range of immune-related pathways, including chemokines, cytokines, and interleukin receptor pathways (Fig 5A-F), as well as six immune cell types, such as B cells and T cells (Fig 5G). Known immune-related genes within the ceRNA network also contribute to HIRI (Fig 5H). Finally, we conducted an immune-related ceRNA subnetwork based on these RNAs, which might affect the immune response and inflammatory processes associated with HIRI (Fig 5I).

### Comprehensive assessment of the association between ceRNA subnetwork and immune cells

**Fig 6:**
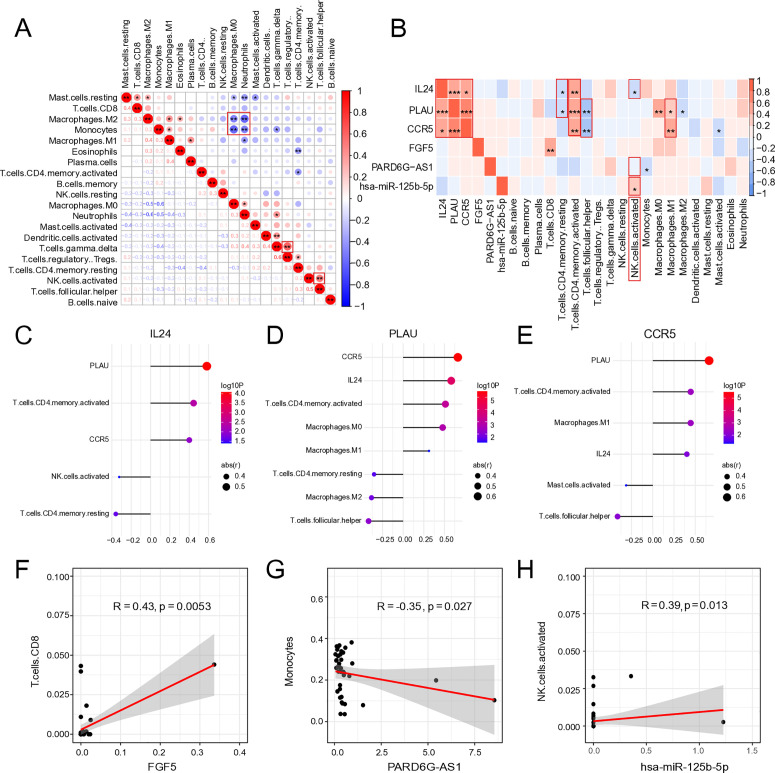
Validation of members of ceRNA subnetwork and immune relevance. A. Spearman analysis of different infiltrating cells and B. the relationships between different tumor-infiltrating cells and members of the ceRNA subnetwork in liver biopsies. C. Showing the relationship between infiltrating immune cells and IL24, D. PLAU, and E. CCR5. F. Further illustrates the exact relationship between FGF5 and CD8 T cells, G. lncRNA PARD6G-AS1 and Monocytes, and F. miRNA has-miR-125b-5p and activated NK cells.

To evaluate the immunological relevance of the ceRNA subnetwork components, we first estimated the content of 22 infiltrating immune cell types from IRI+ and IRI- patients after liver transplants. The interrelationships among these immune cell populations are illustrated in Fig 6A. M2 macrophages and monocytes were significantly negatively correlated with M0 macrophages (R = −0.6, R = −0.5, respectively) and neutrophils (R = −0.5, R = −0.6, respectively). T follicular helper cells were positively correlated with activated NK cells (R = 0.5), and gramma delta T cells were significantly positively correlated with regulatory T cells (R = 0.6).

Subsequently, we examined the interactions among members of the ceRNA subnetwork and correlations with the infiltration of 22 immune cell types. The analysis revealed significant synergistic regulatory relationships both among ceRNAs themselves and between ceRNAs and specific immune cell populations (Fig 6B). Notably, *IL24*, *PLAU*, and *CCR5* were found to interact at the protein level within PPI network, and their expression levels were significantly positively correlated. All three genes exhibited significantly positive correlations with the abundance of activated CD4 ⁺ memory T cells. In contrast, *IL24* and *PLAU* expression levels were significantly negatively correlated with resting CD4 ⁺ memory T cells. Both *PLAU* and *CCR5* expression were significantly positively associated with M1 macrophage infiltration and negatively associated with T follicular helper cells. Additionally, *IL24* expression showed a significant negative correlation with activated NK cells (R = −0.33, p = 4.09e-02, Fig 6C). *PLAU* expression was significantly positively correlated with M0 macrophages, but negatively correlated with M2 macrophages (Fig 6D). *CCR5* expression exhibited a significant negative correlation with activated mast cells (Fig 6E). *FGF5* expression was significantly positively correlated with CD8 ⁺ T cells (Fig 6F), while lncRNA *PARD6G-AS1* showed a negative correlation with monocyte infiltration (Fig 6G). Furthermore, *hsa-miR-125b-5p* was significantly positively associated with activated NK cells (Fig 6H). Interestingly, *IL24* and *hsa-miR-125b-5p* exhibited opposing correlations with NK cell activity, with both *IL24* and *PARD6G-AS1* showing negative associations with NK cell infiltration, indicating that *PARD6G-AS1*-*has-miR-125b-5p*-*IL24* might affect the NK cell activity as a ceRNA sponge (Fig 6B). Collectively, the lncRNA-miRNA-mRNA ceRNA network may play a regulatory role in enhancing or suppressing immune cell infiltration to mediate hepatic ischemia-reperfusion injury.

## Discussion

Recent advancements in high-throughput sequencing technologies and bioinformatics have enabled a comprehensive characterization of the HIRI-associated ceRNA network, as well as its interactions with immune-related signaling pathways and immune cell infiltration. In this study, we identified a potential ceRNA network associated with HIRI and delineated a subnetwork that may modulate immune responses during HIRI. While a limited number of studies have previously reported specific ceRNA axes involved in HIRI, such as SNHG1-miR-186-5p-YY1 [32] and AK054386-miR-199 [12], our work represents the first comprehensive prediction of a ceRNA network specifically implicated in the immune regulatory mechanisms of HIRI. IRI could further enhance innate activation, adaptive immune responses, and cell death programs [1]. Understanding the molecular and immunological consequences of IRI may contribute to novel therapeutic strategies for individuals suffering from IRI-related tissue inflammation and organ dysfunction.

In this study, we identified a ceRNA regulatory subnetwork composed of PARD6G-AS1, hsa-miR-125b-5p, and four immune-associated mRNAs (FGF5, IL24, CCR5, and PLAU) that potentially contribute to HIRI. Immune association analysis revealed that hsa-miR-125b-5p was related to pathways including chemokines, cytokines, and interleukin receptor. Additionally, PARD6G-AS1 was related to the cytokine pathway associated with immune cell populations, including B and T cells. IL24, CCR5, FGF5, and PLAU were known immune-related genes from the Immport database. Although no literature has shown so far that PLAU, FGF5, and IL24 affect HIRI by regulating immune cell infiltration, studies have reported the important regulatory roles of these two genes in the immune microenvironment and immune response in liver disease [33,34]. Notably, CCR5 has been reported to facilitate HIRI resolution by promoting macrophage migration [35]. Its ligand has been illustrated to elicit a series of early events by chemoattracting macrophages, leading to aggravative inflammation in HIRI [36]. Additionally, these immune-related pathways and immune cell types have been reported to play critical roles in the pathogenesis of HIRI. For example, previous studies have reported that reduction of chemokines such as *CXCL1*, *CXCL2*, and *CXCL6*, along with proinflammatory cytokines *IL-6* and *TNF-α* levels, may significantly inhibit neutrophil recruitment to the liver during IRI in mice [37]. Chemokines, cytokines, and other factors were generated during the acute phase of graft injury after liver transplantation, collectively contributing to developing inflammation and activating both innate and adaptive immune responses [38]. The pathogenesis of HIRI was reported to be complex and influenced by multiple factors, including inflammation, damage-associated molecular patterns, and innate immune response [39]. Interleukin-1 receptor antagonist (*IL-1Ra*), known as a natural anti-inflammatory molecule, which was highly expressed in a variety of inflammatory diseases and plays a key role in the inflammatory occurrence and progression [40,41]. Several studies have suggested that *IL-1Ra* has a potential protective effect for inflammation [42]. Furthermore, a recent study demonstrated that the *NRF2* signaling in CD4 + T cells improved liver transplant outcomes by modulating T cell activation and differentiation, indicating a novel cytoprotective sentinel role of CD4 + T cells-intrinsic *NRF2* signaling in against IR-stress in orthotopic liver transplantation recipients [43]. B-cell lymphoma-2 homology3 (BH3)-only proteins have been identified as key mediators of HIRI in both lean and steatotic livers [44].

Within the ceRNA subnetwork, hsa-miR-125b-5p was significantly downregulated in the IRI+ group after liver transplantation, suggesting a potential loss of regulatory control within this axis. Several molecular strategies may be employed to modulate this network. Restoration of miR-125b-5p using miRNA mimics could re-establish post-transcriptional regulation of its target genes. In parallel, PARD6G-AS1 could be silenced using antisense oligonucleotides (ASOs) or siRNAs to relieve its sponging effect, thereby enhancing miR-125b-5p activity. The effector genes IL24, FGF5, CCR5, and PLAU, which may mediate downstream inflammatory responses, represent additional therapeutic targets. These genes could potentially be downregulated via RNA interference or CRISPR interference, and in the case of CCR5, pharmacological inhibition using existing agents like maraviroc offers a feasible translational approach. While these interventions remain hypothetical, they provide a conceptual framework for future therapeutic exploration. Additional in vitro and in vivo validation will be necessary to determine their functional relevance and therapeutic potential in the context of HIRI.

A limitation of this study is the relatively small sample size, which poses challenges when applying multiple testing correction methods, such as the False Discovery Rate (FDR). In our analysis, we found that applying FDR correction resulted in a highly stringent threshold, with no genes remaining significant. This could potentially exclude biologically relevant genes from our analysis. Consequently, to ensure that potentially important genes were not overlooked, we chose to use unadjusted p-values for gene selection, accepting the trade-off of a higher false positive rate. Additionally, this study lacks comprehensive clinical metadata, such as age, sex, and immunosuppressive treatment information. These factors may act as potential confounders influencing gene expression profiles. However, the transcriptomic datasets used in this study were obtained from publicly available sources where such clinical information was incomplete or unavailable. To mitigate these, we further validated the identified genes through functional enrichment analysis, which reinforced their biological relevance despite the absence of FDR correction and the exclusion of potential clinical confounders.

Another major limitation of this study is the use of bulk RNA-seq data, which captures averaged gene expression across all cell types within liver tissue. This inherently limits our ability to resolve cell-type-specific ceRNA interactions, as ceRNA regulation requires co-expression of lncRNAs, miRNAs, and target mRNAs within the same cellular context. Given the heterogeneous nature of liver tissue, including hepatocytes, Kupffer cells, endothelial cells, and infiltrating immune cells, which limitation may affect the interpretation of ceRNA activity. To partially address this, we integrated immune infiltration analysis to capture cell composition dynamics. However, our immune cell deconvolution analysis utilized the CIBERSORT LM22 reference matrix, which was developed based on peripheral blood mononuclear cells and does not specifically include liver-resident immune subsets such as Kupffer cells. As a result, some liver-specific cell types may have been underrepresented or misclassified in our analysis. While CIBERSORT remains widely used for comparative analyses in bulk transcriptomic studies, future investigations incorporating liver-specific deconvolution models or single-cell RNA sequencing data will be necessary to more accurately capture the unique immune landscape of liver tissue.
